# Clinical similarities among bradykinin-mediated and mast cell-mediated subtypes of non-hereditary angioedema: a retrospective study

**DOI:** 10.1186/s13601-015-0049-8

**Published:** 2015-02-04

**Authors:** Karlijn J G Schulkes, Mignon T Van den Elzen, Erik C Hack, Henderikus G Otten, Carla A F M Bruijnzeel-Koomen, André C Knulst

**Affiliations:** Department of Dermatology and Allergology, University Medical Center Utrecht (G02.124), PO Box 85.500, Utrecht, 3508 GA The Netherlands; Laboratory of Translational Immunology, University Medical Center Utrecht, Utrecht, the Netherlands

**Keywords:** Angioedema, Drug-associated, Idiopathic, Wheals

## Abstract

**Background:**

Non-hereditary angioedema (non-HAE) is characterized by local swelling due to self-limiting, subcutaneous or submucosal extravasation of fluid, and can be divided into three subtypes. These subtypes are believed to have different pathophysiological backgrounds and are referred to in recent guidelines as bradykinin-mediated (e.g. caused by angiotensin-converting-enzyme-inhibitors), mast cell-mediated (e.g. angioedema with wheals) or idiopathic (cause unknown). Bradykinin-mediated subtypes are more closely related to hereditary angioedema than the other forms. Because clinical features of these non-HAE subtypes have not been studied in detail, we have looked at the clinical characteristics of symptoms and potential differences in clinical presentation of bradykinin-mediated and mast cell-mediated angioedema (AE) subtypes.

**Methods:**

A questionnaire was sent to patients presenting with AE at our tertiary outpatient clinic to document clinical characteristics, potential triggers and location of AE. The severity of AE attacks was analysed using visual analogue scales (VAS).

**Results:**

The questionnaire was returned by 106 patients, of which 104 were included in the analysis. AE with wheals, idiopathic AE, and drug-associated AE occurred in 64 (62%), 25 (24%) and 15 patients (14%) respectively. Most patients (62%) reported prodromal symptoms while 63% reported multiple locations for an attack. Face and oropharynx were the main locations of AE attacks of any subtype while swelling was the symptom most frequently reported as severe. Overall severity of the last attack was indicated as severe by 68% of the patients. There were no differences between the subgroups.

**Conclusion:**

This similarity in clinical presentation raises the possibility that ACEi-induced, mast cell-mediated and idiopathic AE share common pathways.

**Electronic supplementary material:**

The online version of this article (doi:10.1186/s13601-015-0049-8) contains supplementary material, which is available to authorized users.

## Background

Angioedema (AE) is caused by a rapid local increase in permeability of capillaries and venules with subsequent extravasation of fluid into the interstitial space, which becomes clinically manifest as self-limiting, localized subcutaneous or submucosal swellings. AE is classified into several subtypes [1-3]. The first step in the classification is to differentiate AE with wheals from AE without wheals. AE with wheals can be diagnosed as chronic spontaneous urticaria (CSU) or chronic inducible urticaria (CINDU), and is presumably mast-cell mediated [2], although treatment with (high doses of) antihistamines does not always lead to complete symptom relief [4]. AE may occur in all forms of CSU and CINDU, except dermographism [3]. It can be caused or aggravated by medical drugs such as NSAIDs and antibiotics [2,5]. AE without wheals can be classified further into hereditary and acquired types. Both can be caused by a C1-inhibitor deficiency, in which case a diagnosis of hereditary AE (C1-INH-HAE) or acquired AE (C1-INH-AAE) can be made. HAE can subsequently be divided in to three types, C1-INH-HAE types I and II caused by C1 inhibitor deficiency and hereditary AE with factor XII mutations or of unknown origin (formerly known as type III HAE), which causes enhanced generation of bradykinin [1,6].

AE without wheals can also be associated with the use of angiotensin-converting-enzyme-inhibitors (ACEi). ACEi causes AE which is presumably bradykinin-mediated and is more closely related to hereditary angioedema (HAE) than the other forms [6]. Finally, idiopathic AE is diagnosed when all other causes have been excluded [2-6]. Idiopathic AE can be either histaminergic or non-histaminergic, based on the response to antihistamines [6]. It is unclear to what extent idiopathic AE has a similar pathogenesis to angioedema in patients suffering with chronic spontaneous urticaria (CSU).

Clinical characteristics for HAE are well-described in previous literature [1,7]. For non-HAE however, symptoms and clinical impact are not well described. In this study, a large unselected group of non-HAE patients was categorized into the three AE subtypes: AE with wheals (mast-cell mediated), ACEi-induced AE (bradykinin-mediated) and idiopathic AE (unknown cause). The clinical characteristics, locations and impact of the disease for each subtype were documented. In addition, we adapted the VAS tools developed for HAE and supplemented them with extra symptom scores, and used these to assess severity and type of symptoms of the last AE attack in these patients [8,9].

## Methods

### Patients

All patients visiting the outpatient clinic of the Department of Dermatology and Allergology of the UMC Utrecht between October 2007 and December 2010 for evaluation of angioedema were selected. The diagnosis AE was based on a history of bouts of mucocutaneous or subcutaneous swellings. All case records were checked by one of the investigators to verify the diagnosis. Exclusion criteria were (a) decreased C4-value or proven HAE or AAE due to C1-inhibitor deficiency; (b) patients known to have comorbid malignancy requiring active treatment, because we wanted to avoid any unnecessary discomfort for patients with this disease; and (c) incapability of a patient to fill out the questionnaire. Four patients with AE were excluded from the study because they met one of these criteria, 2 with malignancy, 1 with a cerebrovascular accident and 1 with psychiatric disease.

This study was approved by the ethics committee of the UMC Utrecht, protocol number 13-241/C.

### Questionnaires

Questionnaires were sent to all selected patients to evaluate the subtype and characteristics of their AE. A written reminder was sent to patients who failed to reply after 2 weeks. Two weeks after the first reminder patients who had still failed to respond were contacted by phone and asked to complete the questionnaire. Of the 165 patients to whom the questionnaire was sent, 106 (64%) returned it. One patient did not meet inclusion criteria and the remaining 105 patients were included in the study. Reasons for not filling out the questionnaire included: lack of time (n = 15), lack of a recent AE attack (n = 16), as ascertained during a phone interview, or were unknown (n = 28).

### Evaluation of symptoms and locations

The questionnaire consisted of 17 general questions related to the frequency and impact on daily life, locations involved and treatment of AE attacks. The questionnaires were provided with images to mark the location (s) involved during the last attack. We elected to evaluate the last attack rather than, for instance, the most severe one in order to minimise recall bias. Moreover, we restricted the analysis to patients who had visited the clinic within the recruitment period. In addition, the questionnaire was designed in such a way that answers were double-checked whenever possible. For example, location of the last attack by questionnaire was verified by asking patients to indicate this location also on graphs.

### Severity of symptoms

Furthermore the questionnaire contained a series of symptom-specific visual analogue scale (VAS) [6,7] to assess the severity of the last attack of AE. A value of <20 mm was considered to represent a symptom or attack of minimal severity, >20 mm-50 mm a moderate symptom or attack, and >50 mm a severe symptom or attack. Different sets of VAS [7] were used and expanded for different anatomical locations of an AE attack; namely the face (eyelids, cheeks, lips and ears), the oropharyngeal cavity (tongue, throat, uvula and vocal cords), the extremities (arms, hands, legs, feet and trunk (also referred to as peripheral locations) and the abdomen.

### Subtyping of AE

The following, clinically defined, subgroups of AE were discriminated in patients who had completed the questionnaire:

1. AE associated with wheals, (AE with wheals): Patients were included in this category when they had associated wheals or pruritus alone or if they scored at least 20 mm on a VAS for pruritus when scoring the severity of their last AE attack. In case of a relation with NSAID or ACEi use they were considered to be drug-associated (see next subgroup).
2. AE associated with the use of drugs as NSAID, antibiotics or ACEi: Patients were included in this category when AE became manifest for the first time after using these drugs. As we did not identify patients with AE induced by NSAIDs or antibiotics, this subgroup is further referred to as ACEi-induced AE.
3. AE associated with other diseases such as auto-immune disease: This category is further referred to as AE due to other causes. After subtyping, this subgroup resulted in only one patient with AE related to food exposure, who was excluded from further analysis as the group was not sufficiently large to produce statistically reliable results.
4. Idiopathic AE was diagnosed when no other cause for AE could be identified.

### Data management

Differences between the different subgroups were evaluated by analyzing the answers to the general questions in the questionnaire and the VAS scores of the last AE attack. The VAS forms were used to assess the location as well as the severity and type of symptoms. Analysis of the time to 50% reduction and to minimal symptoms of the last attack was carried out using the answers to that specific question.

All patients who completed and returned the questionnaire were allocated to subgroups. After exclusion of the patient in the group of AE due to other causes, the final number of patients, 104 in total, were included in the analysis. Descriptive statistics were used to describe the data. The data was presented as median values with interquartile ranges.

## Results

### Patient characteristics and subtypes of AE

The median age of the 104 patients included in the study was 55 years and 67 (64%) were female. The median age of onset of AE was 46 years. 12 patients had a positive family history of AE, but none had a proven C1-inhibitor deficiency. 64 patients (62%) had AE with wheals, 15 (14%) had ACEi-induced AE, and 25 (24%) had idiopathic AE. Demographic data of the different subgroups is listed in Table 1.

**Table 1:** **Clinical characteristics of nonHAE patients (n-104)**

	**Total group n = 104**		**Idiopathic n-25**		**AE with wheals n = 64**		**ACEi-induced n-15**	
Male (%)	37	(36%)	14	(56%)	15	(23%)	8	(53%)
Age (years)	55	(42–65)	61	(44–67)	50	(40–61)	64	(58–67)
Family history with AE	12	(12%)	1	(4%)	11	(17%)	0	(0%)
Family history unknown	15	(14%)	4	(16%)	7	(11%)	4	(27%)
Age of onset	46	(35–60)	47	(36–61)	41	(33–54)	59	(63)

Data is presented as numbers and percentages or median values with interquartile ranges.

### Locations of AE attacks

**Figure 1:**
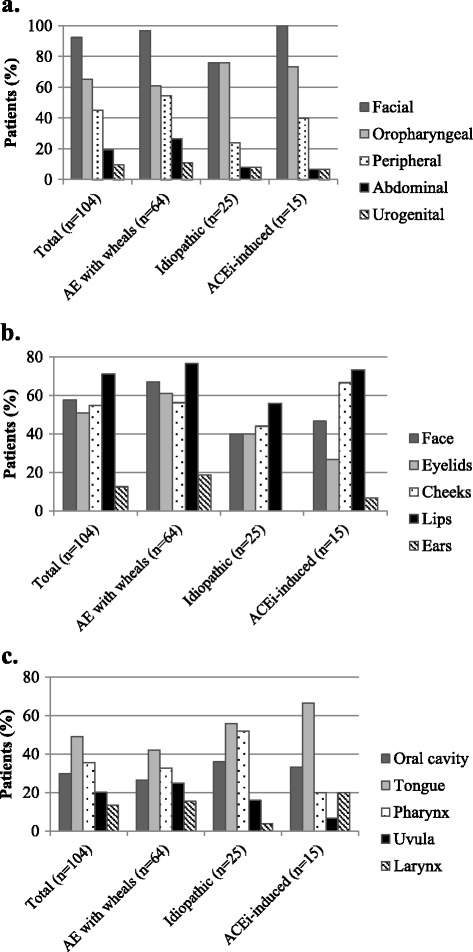
**Locations of AE attacks.** Reported locations of attacks for the total group (n = 104) and by subtype of angioedema for all locations, presented as percentages **(a)**. A breakdown of facial **(b)** and oropharyngeal attacks **(c)** is also presented. The exact numbers of all locations, by subtype, are available as additional file.

All reported locations are shown in Figure 1a. In all subgroups, the face was mentioned most frequently as a location of attacks (96 [92%]). The second most frequent location for all subgroups was the oropharynx (68 [65%]). 47 (45%) mentioned a previous peripheral attack. 10 (10%) mentioned at least one attack in the genital location, and 20 (19%) reported at least one attack in the abdominal location. Figure 1b shows a breakdown of the frequency of facial attacks and Figure 1c shows a breakdown of the frequency of oropharyngeal locations of AE attacks. The lips are the most frequently involved location in the face, while the ears are the least involved. No patients in the idiopathic AE subgroup reported an attack of the ears. The tongue and pharynx were the most frequent locations of attacks in the oropharynx, with 49% and 35% of all patients suffering with attacks at these locations, respectively. Laryngeal attacks occurred in 13% of patients. Laryngeal locations were less frequent in the idiopathic AE subgroup (1 patient (4%) with idiopathic AE) as compared to 15% for AE with wheals and 20% with ACEi-induced AE. In ACEi-induced AE, uvular locations were less frequent (7%) compared to idiopathic AE (16%) and AE with wheals (25%). All different anatomical locations involved in at least one historical attack mentioned by the patients are shown in Additional file 1.

Sixty-six of the 104 patients (63%) reported that they had suffered AE attacks at multiple locations. Patients with idiopathic AE reported this less frequently (up to 40% of all attacks were at multiple locations) than the other groups. Characteristics of angioedema attacks are summarized in Table 2. 62% of the patients reported prodromal symptoms preceding an attack of AE. This included paraesthesia, pruritus or erythema. Only 40% of the patients with ACEi-induced AE reported prodromal symptoms (n = 6, of which n = 3 reported pruritus).

**Table 2:** **Characteristics of non-HAE attacks per patient (n = 104)**

*Prodromal symptoms*	64 (62)*
Pruritus	17 (16)
Paresthesia	15 (14)
Erythema	1 (1)
Other	30 (28)
*Multiple locations*	66 (63)
*Duration of attacks*	*50% improved*
Face	23**
Oropharynx	15.2
Peripheral	30.3
Abdominal	8.7
	*100% resolved*
Face	57**
Oropharynx	37.8
Peripheral	66.6
Abdominal	33
*Frequency of attacks*	
<1 per year	14 (13)*
>1 per year but <1x per month	37 (36)
>1 per month but <1x per week	21 (20)
>1 per week	19 (18)
Daily	4 (4)
Unknown	9 (9)

*Data is presented as numbers and percentages; **data presented as median hours.

### High severity and long duration of the last AE attack

**Figure 2:**
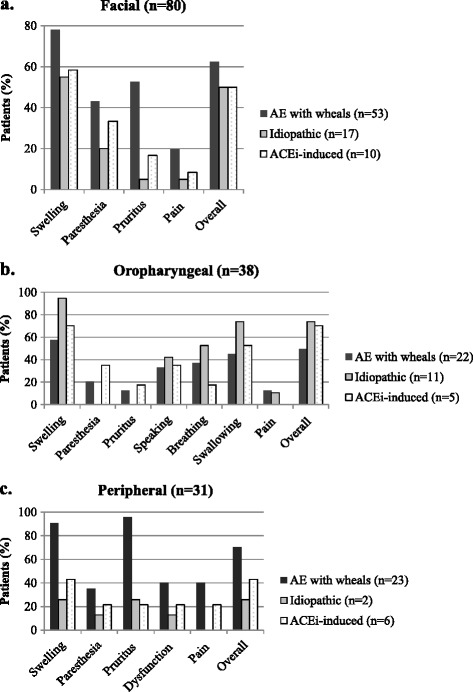
**Symptoms per location of AE attacks.** Symptoms of the last angioedema attack of each patient reported to be severe for facial **(a)**, oropharyngeal **(b)** and peripheral locations **(c)**. Percentages on the Y-axis represent the percentage of patients that reported the indicated symptom VAS score as ≥50 mm. Note that the number of patients varies between the different locations because the location of the last attack differs between patients.

VAS scores for the last AE attack were completed by 99 of the 104 patients. 67 (68%) rated the overall severity of the last attack as ≥50 mm on VAS for at least one involved location. For facial and oropharyngeal locations, swelling was the most frequent symptom reported as ≥ 50 mm (Figure 2a and b). In addition, many patients with oropharyngeal AE reported severe (VAS ≥50 mm) difficulties in swallowing (55%), severe changes in speech (41%) and severely impaired breathing (37%). Pruritus and swelling were the symptoms most frequently scored severe for peripheral AE (Figure 2c). Ten patients with peripheral AE also reported severe pain , but this was not reported by idiopathic AE with peripheral locations. VAS scores did not differ between AE subgroups for oropharyngeal attacks. Median time to 50% resolution was 23 hours for facial attacks, 15 hours for oropharyngeal attacks, 30 hours for peripheral attacks, and 9 hours for abdominal attacks (Table 2). Median time to complete resolution was 57 hours for facial lesions, 38 hours for oropharyngeal attacks, 66.6 hours for peripheral attacks, and 33 hours for abdominal attacks.

### Impact of AE attacks on daily life

Of the total group, a minority showed to have a high frequency of attacks: 20 patients (19%) reported having suffered an attack more than once a week, 23 (22%) more than once a month, 32 (31%) more than once a year, and 22 patients (21%) less than once a year. The frequency of attacks was comparable in most subgroups except that, compared to other subgroups, in the idiopathic group more patients reported having suffered an attack less than once a year (32% versus 17-20%, respectively).

Medical burden and social impact on daily life of having AE are summarized in Table 3. Of all 104 patients, 22 (21%) reported having sought medical advice when suffering with an AE attack. Twenty-nine patients (28%) reported having been admitted to the hospital at least once because of an AE attack of which 5 (5%) had been admitted to an Intensive Care Unit at least once. A majority of the patients (77%) reported having used antihistamines on demand during an AE attack. Especially in AE with wheals, oral corticosteroids were used on demand as well. Sixty-one (59%) used antihistamines as prophylactic medication, 29 patients (28%) reported prophylactic use of oral corticosteroids to prevent attacks of swelling.

**Table 3:** **Medical burden and social impact of angioedema (n = 104)**

		Total group n = 104*	AE with wheals n = 64*	Idiopathic n = 25*	ACEi-induced n = 15*
*Medical burden*					
Seek medical advice	Yes	22(21)	13(20)	7(28)	2(13)
	Unknown	4(4)	2(3)	2(8)	0(0)
Angioedema-related admission	To hospital	29(28)	19(30)	5(20)	5(33)
	To ICU	5(5)	2(3)	2(8)	1(7)
*Social impact*					
AE-related absenteeism	<1 per year	60(58)	36(53)	16(64)	10(67)
	>1x per year but <1x per month	19(18)	12(19)	6(24)	1(7)
	>1x per month but <1x per week	5(5)	4(6)	1(4)	0(0)
	>1x per week	3(3)	3(5)	0	0
	Not applicable	15(15)	9(14)	2(8)	3(20)
	Unknown	2(2)	2(3)	0(0)	1(7)

*Data are presented as numbers and percentages: n (%).

Three of 104 patients (3%) reported having been absent from work or school because of an AE attack more than once a week, 5% more than once a month, 19 (18%) more than once a year and 61 (59%) less than once a year. 15 could not be evaluated in this respect since they neither had a job nor went to school. Surprisingly, absenteeism was seen mostly in patients with a lower attack frequency, possibly indicating that patient with frequent symptoms accept symptoms to some extent.

## Discussion

To our knowledge, this is the first study to describe in detail the characteristics of angioedema attacks in a large group of non-hereditary angioedema patients, and to study potential differences between clinical subtypes. In contrast to previous literature [10], we also included information of patients suffering with AE in the presence of wheals. It has been suggested previously that angioedema resulting from bradykinin release and resulting from mast cell mediator release might show similar signs and symptoms [2,11]. In this study we show that clinical manifestations of attacks in patients with non-HAE subtypes are indeed remarkably similar in locations, frequency and severity of attacks. Our 104 patients could be allocated to the following, clinically relevant subtypes of AE: AE with wheals (n = 64), ACEi-associated (n = 15), AE due to other causes (n = 2, both excluded from analysis) and idiopathic AE (n = 25). Of these subtypes, AE with wheals is believed to be mast cell-mediated, idiopathic AE may be either mast cell-or bradykinin-mediated and ACEi-associated AE is believed to be bradykinin-mediated. Beltrami et al. reported on 111 patients with ACE-inhibitor angioedema. After discontinuation of the ACE inhibitor, 46% of patients had further recurrences of angioedema, although less-frequent. These findings suggest that ACE inhibitors may certainly exacerbate angioedema in a large subset of patients but may not be the sole cause of angioedema [12].

All patients reporting pruritus, itching of AE lesions or presence of both AE and wheals, were allocated to the AE with wheals subgroup when they did not use ACEi or NSAIDs. In drug-associated AE, ACEi, antibiotics and NSAIDs are commonly known culprits [13]. It is arguable that NSAID-induced AE and ACEi-induced AE should be combined in a single subtype, as the first seems to be mast cell mediated and the latter bradykinin mediated. In our study this has no effects on the data as there were no patients included with AE triggered by NSAIDs. This classification is based on etiological features. It is deliberately not based on biochemical mechanisms because of a lack of evidence on this topic. These subgroups are in line with previous literature [2-4,6], although different classifications are sometimes used [14].

We did not find striking differences among the demographic parameters of AE subtypes, except for a higher median age of ACEi-induced AE. This finding most likely reflects the fact that patients taking medication, especially antihypertensive drugs, are generally older.

The majority of our patients (62%) reported prodromal symptoms whereas in previous surveys among HAE patients even higher percentages ranging from 82.5-95.7% were reported [15]. Strikingly, 50% of the reported prodromal symptoms in ACEi-induced AE consisted of pruritus, which would not be expected for a bradykinin-mediated swelling. Our study was not designed to determine the interval between the reported prodromal symptoms and the onset of angioedema. To our knowledge, no study has been performed to measure prodromal symptoms in chronic urticaria patients. Also, a majority of patients reported involvement of multiple locations during attacks (63%). Face and oropharynx were the most frequent locations. AE attacks involving the extremities had the longest resolution time (almost 67 hours). We did not find gross differences in involved locations and symptoms among the various AE subtypes. For all subtypes most AE attacks occur in the face and in the oropharynx, which is consistent with recent published literature [16]. In HAE due to C1-inhibitor deficiency, extremities and the gastrointestinal tract are preferred locations [9,17-20]. In contrast, in HAE with normal C1 inhibitor [21], attacks also occur most frequently in face and oropharynx. One may therefore speculate that C1-inhibitor deficiency per se influences the location of AE attacks, though the molecular mechanism to explain this effect of C1-inhibitor is far from clear. Another option is that in non-HAE, abdominal attacks are not as well recognized as in HAE leading to underreporting.

63% of the patients reported lesions on multiple locations during a single attack. Such a high frequency of multiple locations has also recently been reported in a study on peripheral attacks of HAE [9]. This might indicate that AE attacks result from a systemic trigger, rather than a local activation process. However, it cannot be excluded that local activation of biochemical processes occurs at multiple sites at the same moment. This is one of the topics in angioedema that should be explored further in future research.

Our study is retrospective in nature and therefore may have limitations due to recall bias. We tried to minimize this (as described in the methods section of this article), however the recall period of 5 years is rather long. In the vast majority of patients (83%), the last attack was reported less than two years in the past. Another limitation is the number of missing values of C1-INH or C4. For idiopathic AE, blood test results were missing in 58% of patients, for AE with wheals this was the case in 48% of the patients. However, in all patients where blood tests were performed, the test results were negative. We suspect this had only a limited impact on our results.

Furthermore, patients reporting pruritis were allocated to the subgroup AE with wheals. However, pruritis was also reported as a prodromal symptom. It may be difficult to separate pruritus as a prodromal symptom from pruritus as a symptom in patients with wheals. This could lead to overestimation of the AE subtype with wheals. Additionally, the ACEi-induced AE group may be underestimated due to missing values in the medical records, causing a possible overestimation of the idiopathic AE group. We think this had limited impact on our conclusions since no major differences were observed between the subgroups.

VAS scoring provides a sensitive, reliable and validated tool for evaluation of patient reported outcome measures such as pain [22-25]. We used VAS scoring to assess severity of pain as well as that of other symptoms of the AE attacks. This analysis revealed that swelling is the dominant symptom of AE independent of subtype. Strikingly, the patients reported a high proportion (69%) of severe attacks. There is literature that suggests that VAS scores can be obtained retrospectively. In a previous study, VAS-scores were validated as an instrument for measuring HAE attack severity. VAS scores were obtained retrospectively. HAE patients reported VAS scores as if they were experiencing an acute angioedema attack at the time [26]. We feel that the use of VAS in retrospect is competent, however, as stated earlier, the recall period is rather long in our study.

Our study was not designed to evaluate the efficacy of treatment in the AE subtypes. Interestingly, 79% of the patients reported having not sought medical help in case of an acute AE attack. This is remarkable given that 65% of the patients rated their last attack as severe. Our study was not designed to evaluate the reasons why patients are reluctant to seek medical advice in case of an acute AE attack. However, one may speculate that patients with AE underestimate the severity and potential consequences of their disease. We have made similar observations in food allergic patients [27].

We conclude that despite different etiologies, there are strong clinical similarities among different subtypes of non-HAE. Except for age, we did not find striking differences between mast cell-mediated (AE with wheals and idiopathic AE) and bradykinin-mediated AE (ACEi-induced AE), with regards to dominant symptoms, preferred locations, and prodromal symptoms. These findings support the previous suggestion that angioedema resulting from bradykinin release and resulting from mast cell mediator release show similar signs and symptoms [2,11].

Further research should address the question whether or not these subtypes of non-HAE share a final common biochemical pathway leading to non-HAE since our questionnaires were not designed to study this. Identification of the molecular mechanisms of this pathway may provide new targets for future intervention in all non-HAE subtypes.

### Consent

The study was not covered by the Medical Research Involving Human Subjects Act which was confirmed by the Medical Research Ethics Committee of the UMC Utrecht, the Netherlands. Written informed consent for the publication of this report and any accompanying images was not required from the patients as approved by the Ethics Committee.

## Additional file

Additional file 1:
**All different anatomical locations involved in at least one historical attack mentioned by the patients.**

## Abbreviations

ACEi: Angiotensin-converting-enzyme-inhibitors
AE: Angioedema
CINDU: Chronic inducible urticaria
CSU: Chronic spontaneous urticaria
HAE: Hereditary angioedema
ICU: Intensive care unit
NSAID: Non-steroidal anti-inflammatory drugs
VAS: Visual analogue scale
