# Supplementary material for: Clinical similarities among bradykinin-mediated and mast cell-mediated subtypes of non-hereditary angioedema: a retrospective study
Source: Clin Transl Allergy. 2015 Feb 4;5(1):5. doi: 10.1186/s13601-015-0049-8 (PMC4320604; doi:10.1186/s13601-015-0049-8)
Supplement: Additional file 1: — All different anatomical locations involved in at least one historical attack mentioned by the patients. [file 13601_2015_49_MOESM1_ESM.docx]

**Additional file 1:**

All different anatomical locations involved in at least one historical attack mentioned by the patients

| Location | | Urticaria-associated (n=64) | | Idiopathic (n=25) | | ACEi-induced (n=15) | | Total  (n=104) | |
| --- | --- | --- | --- | --- | --- | --- | --- | --- | --- |
|  |  | n | *(%)* | n | *(%)* | n | *(%)* | n | *(%)* |
| Facial | Face | 43 | *67* | 10 | *40* | 7 | *47* | 60 | *58* |
|  | Eyelids | 38 | *59* | 10 | *40* | 4 | *27* | 52 | *50* |
|  | Cheeks | 36 | *56* | 11 | *44* | 10 | *67* | 57 | *55* |
|  | Lips | 49 | *77* | 14 | *56* | 12 | *80* | 75 | *72* |
|  | Ears | 12 | *19* | 0 | *0* | 1 | *7* | 13 | *13* |
|  |  |  |  |  |  |  |  |  |  |
| Oropharyngeal | Oral cavity | 17 | *27* | 9 | *36* | 5 | *33* | 31 | *30* |
|  | Tongue | 27 | *42* | 14 | *56* | 10 | *67* | 51 | *49* |
|  | Pharynx | 21 | *33* | 13 | *52* | 3 | *20* | 37 | *36* |
|  | Uvula | 16 | *25* | 4 | *16* | 1 | *7* | 21 | *20* |
|  | Larynx | 10 | *16* | 1 | *4* | 3 | *20* | 14 | *13* |
|  |  |  |  |  |  |  |  |  |  |
| Peripheral | Arms | 32 | *50* | 6 | *24* | 4 | *27* | 42 | *40* |
|  | Legs | 28 | *44* | 6 | *24* | 6 | *40* | 40 | *38* |
|  |  |  |  |  |  |  |  |  |  |
| Abdominal |  | 17 | *27* | 2 | *8* | 1 | *7* | 20 | *19* |
| Urogenital |  | 7 | *11* | 2 | *8* | 1 | *7* | 10 | *10* |
